# Angiotensin Receptor Blockade Does Not Decrease Synthetic Angiotensin II (Giapreza^®^) Effectiveness in Perioperative Hypotension Surrounding Kidney Transplant

**DOI:** 10.3390/biomedicines13061442

**Published:** 2025-06-12

**Authors:** Natalie Pettit, Jamie Benken, Benito Valdepeñas, Nishita Gandhi, Rama Alyousef, Scott Benken

**Affiliations:** Retzky College of Pharmacy, University of Illinois, Chicago, IL 60612, USA; nataliep@uic.edu (N.P.); jjosep9@uic.edu (J.B.); bvalde2@uic.edu (B.V.); ngandh23@uic.edu (N.G.); ralyou2@uic.edu (R.A.)

**Keywords:** angiotensin II, antihypertensive agents, kidney transplantation, angiotensin receptor blockers, perioperative hypotension, shock, vasopressors

## Abstract

**Background/Objectives**: The use of angiotensin II (AT2S) as a vasopressor in patients receiving angiotensin receptor blockers (ARBs) prior to kidney transplant (KT) raises theoretical concerns. At our center, AT2S is the first-line vasopressor during KT. This study evaluated the hemodynamic and clinical effects of pre-transplant ARBs on AT2S use in KT. **Methods**: This single-center, retrospective cohort trial included patients with hypertension ≥ 18 years old on antihypertensive therapy who received AT2S as the first-line vasopressor peri-transplant. Patients were divided into ARB and non-ARB cohorts. Primary outcomes included total AT2S duration, time with SBP < 120 mmHg, and need for additional vasopressor support. **Results**: A total of 65 patients were analyzed: 22 in the ARB group and 43 in the non-ARB group. There were no significant differences in the frequency or duration of SBP < 120 mmHg or additional vasopressor requirements between groups (*p* > 0.05). Hospital and ICU stay length, safety, and adverse drug events were also similar. **Conclusions**: Contrary to theoretical concerns and observations in other distributive shock populations, no significant hemodynamic or clinical differences were observed in the response to AT2S in patients with pre-transplant ARB use.

## 1. Introduction

Hypertension is a common comorbid disease state for patients with end-stage renal disease (ESRD) on maintenance hemodialysis, with more than 80% of patients affected [1]. The reasons for hypertension exacerbation in this population include volume overload, arterial stiffness, dysregulated renin–angiotensin–aldosterone system (RAAS) activity, endothelial dysfunction, and the use of erythropoietin-stimulating agents [1]. While there is no consensus on blood pressure goals in this ESRD population, a systolic blood pressure (SBP) goal of less than 130–140 mmHg is generally accepted [2,3,4,5]. Angiotensin receptor-blocking agents (ARBs) are among the first-line pharmacologic medications administered to this population for hypertension treatment [6].

As patients transition from ESRD on the transplant waiting list to receive a kidney transplant, maintaining hemodynamic perfusion to the allograft during transplantation is vital for allograft survival. Hypoperfusion is often associated with hypotension, and hypoperfusion to the allograft has been associated with acute kidney injury (AKI), slow graft function (SGF), delayed graft function (DGF), and allograft loss [7]. Risk factors for intraoperative hypotension include volume loss, anesthesia medications, and the use of chronic antihypertensive medications before transplantation [8]. While not formally defined, studies suggest a goal SBP of ≥120 mmHg to maintain adequate allograft perfusion [9,10,11,12]. When the SBP falls below this threshold perioperatively, it is recommended that the patient be worked up for shock and that the necessary measures be implemented to reverse the most common cause [13]. This may require fluids and inotropic support for hypovolemic or cardiogenic shock, respectively, but it will most often result in the diagnosis of vasodilatory shock, necessitating vasopressor therapy [9]. In our patient population, just under one half of our kidney transplant recipients require vasopressor therapy [14]. Our institutional algorithm for vasopressor support during and after KT utilizes synthetic angiotensin II (AT2S—Giapreza^®^ [Innoviva Specialty Therapeutics, Inc. Burlingame, CA, USA]) as the first-line vasopressor [15].

Theoretical concerns exist when using AT2S as a vasopressor in patients taking ARBs. The AT2S package insert lists ARBs as potential drugs that interact with AT2S [16]. ARBs may decrease the response to AT2S due to the angiotensin II type-1 receptor blockade that occurs with ARB usage [17,18]. This competitive inhibition may dimmish AT2S effectiveness. While data is limited, in the septic shock setting, the AT2S response could be altered when ARBs are present [19]. In the vital intraoperative setting of kidney transplant, even short durations of not meeting hemodynamic goals can significantly worsen renal outcomes [5], making this drug interaction of utmost clinical significance for this population. These drug interactions have not been clinically evaluated in this setting.

This study aimed to evaluate the impact of ARB agents compared to other antihypertensive agents on AT2S effectiveness and safety when used for patients receiving kidney transplant. We hypothesized that patients taking ARBs compared to other antihypertensive therapies before transplant will have decreased hemodynamic response to AT2S used in the perioperative period of transplant.

## 2. Materials and Methods

### 2.1. Study Design and Population

**Figure 1:**
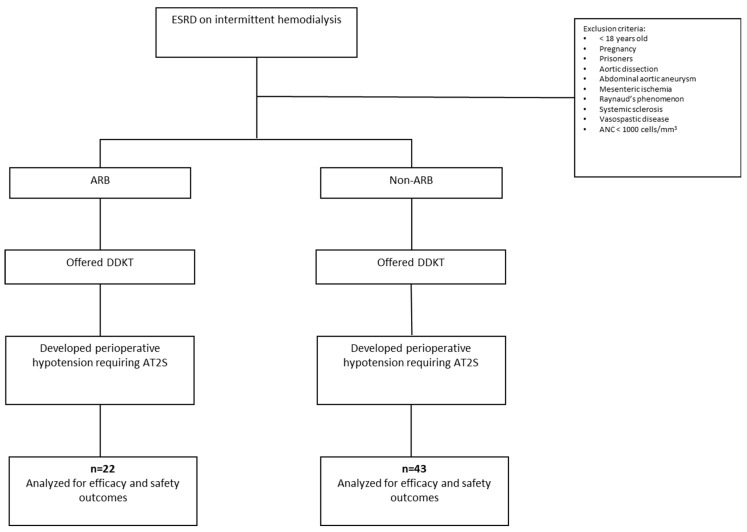
CONSORT diagram for evaluation of patients for inclusion and exclusion. ANC = absolute neutrophil count; ARB = angiotensin II type-1 receptor-blocking agents; AT2S = synthetic angiotensin II; DDKT = deceased-donor kidney transplant; ESRD = end-stage renal disease.

This study is an institutional review board-approved (STUDY2022-1122), single-center, retrospective cohort trial. Patients were included if they were at least 18 years old, were on maintenance hemodialysis, underwent a deceased-donor kidney transplant (DDKT) between 1 January 2022 and 1 January 2024, had hypertension requiring pharmacologic antihypertensive therapy before transplant, and received AT2S for hypotension during the perioperative kidney transplant period. The perioperative period included the intraoperative period and the immediate 24 h after the DDKT. Patients were excluded if they were pregnant, were prisoners, had a history of aortic dissection, abdominal aortic aneurysm, mesenteric ischemia, Raynaud’s phenomenon, systemic sclerosis, or vasospastic disease, or had an absolute neutrophil count of less than 1000 cells/mm^3^ in the 18 months prior to transplant. Patients who were not on maintenance hemodialysis, as well as those taking midodrine or fludrocortisone, were excluded (Figure 1).

Patients were separated into two cohorts, ARB or non-ARB, based on their antihypertensive regimen prior to transplant. Patients in the ARB cohort were taking an ARB and could also be on any other antihypertensive agents listed, excluding angiotensin-converting enzyme inhibitors (ACEis) given their opposing effects on the RAAS. Patients in the non-ARB cohort were taking at least one antihypertensive medication from the following pharmacologic classes: ACEis, calcium channel blockers (CCBs), beta blockers (BBs), loop diuretics, thiazide diuretics, potassium-sparing diuretics, alpha-2 agonists, or direct vasodilators (i.e., hydralazine, nitrates). Antihypertensive medications were identified via the pre-transplant pharmacist note written on the day of transplant and the medication dispensing history available in the electronic medical record (EMR).

**Figure 2:**
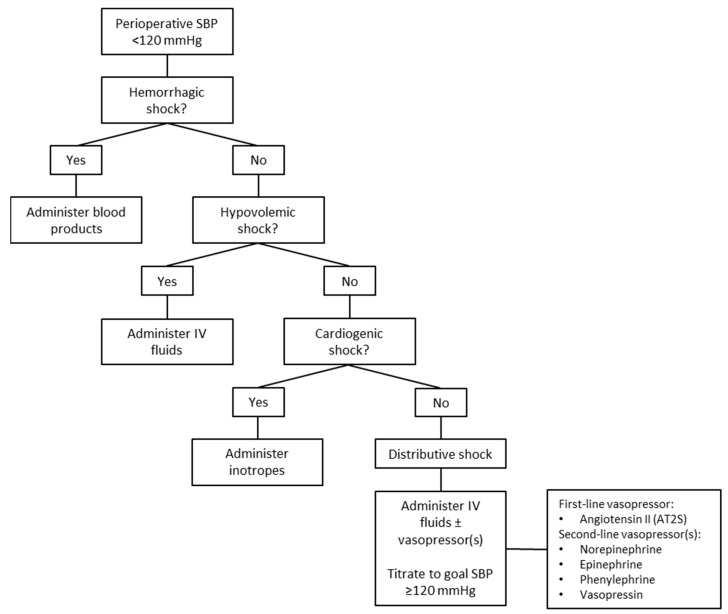
Institutional algorithm for evaluation of perioperative hypotension surrounding kidney transplantation and vasopressor usage. If the blood pressure falls below 120 mmHg, patients are assessed for each shock state, with appropriate resuscitation agents utilized based on the cause of the shock. These could include blood products, intravenous crystalloids or colloids, inotropes, or, for distributive shock, the use of vasopressor agents. IV = intravenous; SBP = systolic blood pressure.

Hypotension during the perioperative KT period was defined according to our institutional protocol as an SBP < 120 mmHg. Our kidney transplant institutional protocol for blood pressure involves a surgeon and an anesthesiologist assessing hypotension due to hypovolemia, either through blood loss or fluid loss, cardiogenic shock, or distributive shock. Hemorrhagic shock is treated with blood products, non-hemorrhagic hypovolemic shock with intravenous fluids, and cardiogenic shock with inotropes (Figure 2). If patients have distributive shock despite intravenous fluids, AT2S is administered as a continuous infusion at a recommended starting rate of 20 ng/kg/min and titrated by 5 ng/kg/min every 5 min as needed to achieve and maintain a goal SBP ≥ 120 mmHg. The maximum rate of AT2S during the first 3 h is 80 ng/kg/min and beyond this period is 40 ng/kg/min. 

Additional vasopressor support (norepinephrine [NE], epinephrine [EPI], phenylephrine [PE], dopamine [DA]) is added as needed for additional blood pressure support at the discretion of the anesthesiologist and surgeon.

### 2.2. Objectives

The primary objective of this study was to compare the hemodynamic effectiveness of AT2S across the ARB and non-ARB cohorts. Hemodynamic effectiveness was defined as a comparison of the total duration of AT2S use, the number of instances with SBP < 120 mmHg, the need for one or more additional continuous vasopressor agents, and time below the goal SBP of 120 mmHg while on AT2S. Hemodynamic parameters were captured at baseline, every 15 min intraoperatively, and every hour postoperatively. Secondary outcomes included safety and length of stay (LOS) in the hospital and ICU. Safety outcomes while on AT2S included cumulative incidence of tachycardia confirmed by electrocardiogram (EKG), thromboembolic events including deep venous thrombosis (DVT) identified on venous duplex ultrasounds, pulmonary embolism (PE) confirmed by computed tomography (CT) angiogram, stroke confirmed by note documentation, and myocardial infarction confirmed by percutaneous intervention. The incidence of an SBP greater than 180 mmHg and hyperglycemia requiring a continuous insulin infusion while on AT2S was also recorded.

To ensure that the patient groups were comparable, we analyzed various factors including patient demographics, comorbidities, antihypertensive agents, and transplant characteristics such as donor age (years), terminal serum creatinine (mg/dL), cold ischemic time (hours), kidney donor profile index (KDPI, %), duration of kidney transplant (hours), first serum creatinine (SCr—mg/dL), and creatinine clearance (CrCl—mL/min) post-transplant. To validate hemodynamic effectiveness, we reviewed other variables that could influence hemodynamics including intravenous (IV) fluid volume (mL), blood loss (mL), blood products administered (mL), push-dose vasopressor characteristics, and propofol use.

### 2.3. Statistical Analysis

Normally distributed continuous variables were summarized using means with standard deviations (SD) and compared using Student’s T-test. Non-normally distributed continuous and ordinal variables were summarized using medians and interquartile ranges (IQR) and compared using the Mann–Whitney U-test. Categorical variables were summarized using counts and proportions and compared using a Chi-Squared test. The patient sample size was driven by AT2S use at our institution; therefore, no a priori calculation was performed. A *p*-value of 0.05 was considered significant for these comparisons. Linear and logistic regression analyses were performed to determine variables associated with the maximum dose of AT2 and with the duration of AT2S usage, respectively. All variables were evaluated with bivariate correlations (e.g., Pearson and Spearman), and variables with significant associations (*p* < 0.1) were included in the regression models as independent variables. A *p*-value of 0.05 was considered significant when evaluating the regression models. All statistical analyses were performed using SPSS version 30.

## 3. Results

A total of 65 patients were analyzed: 22 in the ARB group and 43 in the non-ARB group. Baseline demographics, including age, race, ethnicity, sex, body mass index (BMI), and comorbidities, were similar across groups (Table 1). Transplant-specific characteristics, including KDPI, donor age, and terminal SCr values, were also similar. Patients in the ARB group had a significantly shorter transplant surgery duration of 3.2 ± 5.4 h compared to 5.2 ± 3.1 h in the non-ARB group (*p* = 0.029). Most patients in both groups took more than one antihypertensive medication (17 [77%] ARB vs. 25 [58%] non-ARB, *p* = 0.127), with the ARB group taking 3.4 (IQR = 3) antihypertensive medications vs. 1.8 (IQR = 2) in the non-ARB group (*p* = 0.001). Of the 43 patients in the non-ARB group, 16 (37%) were taking ACE inhibitors. A breakdown of the various antihypertensive agents is summarized in Table 1.

The maximum dose of AT2S was not statistically different between groups (*p* > 0.05, Table 2). In a bivariate correlation for maximum AT2S dose, AT2S duration, ICU duration, ARB exposure, and ACEi exposure were statistically correlated with maximum dose (*p* < 0.1). A linear regression model was created with these variables (F = 9.708, df = 4, *p* < 0.001), and each variable was statistically associated with the maximum dose of AT2. For each hour of increased AT2 duration, there was an increased maximum dose of AT2 by 0.202 ng/kg/min (95% CI 0.086 to 0.318 ng/kg/min; *p* < 0.001). For each hour of ICU duration, there was an increased maximum dose of AT2 by 2.655 ng/kg/min (95% CI 0.531 to 4.779 ng/kg/min; *p* = 0.015). If patients were on preoperative ARBs, they had, on average, a 12 ng/kg/min (95% CI 4.663 to 19.5 ng/kg/min; *p* = 0.002) increase in the maximum AT2 dose. If patients were on preoperative ACEi, they had, on average, a 9.78 ng/kg/min (95% CI −17.48 to −2.037 ng/kg/min; *p* < 0.001) decrease in the maximum AT2 dose.

The average duration of AT2S use was 13.4 ± 15.1 h compared to 26.8 ± 35.1 h in the ARB group and non-ARB, respectively (*p* = 0.267). Additionally, the intraoperative (1.63 ± 1.25 h vs. 1.93 ± 1.67 h, *p* = 0.463) and postoperative (14.3 ± 20.8 h vs. 25.6 ± 36.0 h, *p* = 0.181) AT2S durations were similar between the ARB and non-ARB groups. In a bivariate correlation for AT2S duration, maximum AT2 dose, ICU duration, race, duration of transplant surgery, and ARB exposure were statistically correlated with AT2 duration (*p* < 0.1). A linear regression model was created with these variables (F = 7.005, df = 5, *p* < 0.001), and AT2 maximum dose, transplant duration, and ARB exposure were statistically associated with AT2 duration. For each ng/kg/min of increased AT2 dose, there was an increase in duration of 0.579 h (95% CI 0.135 to 1.023 h; *p* = 0.11). For each hour of transplant surgery duration, there was a decrease in AT2 duration by 2.771 h (95% CI −4.532 to −1.024 h; *p* = 0.002). If patients were on a preoperative ARB, they had, on average, a 16.88 h (95% CI −31.537 to −2.22 h; *p* = 0.025) shorter duration of AT2.

The number of instances with SBP < 120 mmHg, the time with SBP < 120 mmHg, and the need for additional vasopressor support were similar between groups (*p* > 0.05 for all comparisons; Table 2). There was no difference in intraoperative push-dose vasopressor and propofol usage (Table 3). Fluid balance, including estimated blood loss, IV fluids given, and blood products received, was similar (Table 3). Hospital LOS and ICU length of stay were also similar between groups (Table 4). New tachyarrhythmias confirmed by EKG were observed in three (13.6%) patients in the ARB group and four (9.3%) in the non-ARB group, although this finding was insignificant (*p* > 0.05). Insulin infusions for managing hyperglycemia were administered to two patients in each of the ARB and non-ARB groups. No incidence of peripheral ischemia, DVT, pulmonary embolism, myocardial infarctions, or strokes was observed in either group.

## 4. Discussion and Limitations

### 4.1. Discussion

In this retrospective cohort study evaluating the impact of ARBs on AT2S utilization among patients undergoing DDKT experiencing IV fluid-refractory vasodilatory shock, we observed similar hemodynamic effectiveness in patients exposed to ARBs versus other antihypertensive agents as described by the duration of AT2S administration, occurrences and duration of hypotension, and need for additional continuous infusion vasopressor agents. We observed some statistical associations with ARB and AT2S usage, but these observations were inconsistent with the previous literature. The clinical variables of hospital and ICU LOS were comparable between patients receiving ARBs and those not receiving them. Furthermore, there was no discernible variation in the incidence of adverse effects associated with AT2S utilization between the two groups.

While the theoretical drug interaction between AT2S and ARBs has not been the primary focus in clinical studies, small subgroup analyses of larger trials have examined this potential interaction. In a recently published post hoc analysis from the ATHOS-3 trial [19], investigators sought to determine whether prior ARB or ACEi therapy alters the efficacy of AT2S in patients with vasodilatory (primarily septic) shock. In 22 patients who had been exposed to an ARB 7 days prior to randomization, MAP at one hour from the start of AT2S administration was lower compared to patients not exposed to an ARB (−6.0 mmHg, 95% CI −11.5 to −0.6), which was inconsistent with our findings. In our transplant patient group, the hemodynamic outcomes were comparable regardless of whether patients were exposed to ARBs. The inconsistencies in our data may be attributed to the shorter duration of hyperreninemia and/or cytokine release in transplant compared to patients experiencing septic shock, potentially leading to less hemodynamic variability between groups in this study. Furthermore, the ATHOS-3 post hoc study evaluated MAP one hour after initiating AT2S treatment, while our assessment focused on achieving an SBP of ≥120 mmHg, as per our institution’s protocol. Sun et al. concluded that SBP is a more sensitive indicator of early hemodynamic changes, particularly in the perioperative period, compared to MAP [20]. Due to the rapid onset of AT2S, MAP analysis at the one-hour mark may not fully capture hemodynamic differences between groups.

The investigators of the ARAMIS-1 trial found that patients with ARB exposure required higher maximum doses of AT2S than those without ARB exposure [21]. The authors posited that this may be attributed to higher renin levels observed in patients with ARB exposure, which could have led to an alternative metabolic pathway of angiotensin I and angiotensin II, creating vasodilatory byproducts [22,23]. Our study found a similar association with maximum doses of AT2S needed in the ARB group, yet the overall duration of AT2S usage was not impacted. In fact, the opposite effect was observed, with ARB exposure being associated with a shorter duration of AT2 usage. The higher doses of AT2S but shorter duration of AT2S use may have been counterbalanced as the ARB group had a notably shorter duration of kidney transplant surgery, which may be the major contributing factor to vasodilation in this setting. Interestingly, and counterintuitively, the duration of transplant surgery was inversely associated with AT2 duration, potentially leading to the conclusion that there is an unknown factor in the postoperative setting that influences AT2 duration. Additional studies would be needed to refute or confirm these observations.

Both acute and chronic inhibition of angiotensin II type-1 receptors with ARBs leads to increased renin production via the interruption of angiotensin II feedback inhibition [24]. Hyperreninemia leads to increased production of angiotensin I and, subsequently, angiotensin II if possible. In patients exposed to ARBs, during vasodilatory shock, renin and angiotensin I baseline levels were increased in the ATHOS-3 post hoc analysis compared to in those not exposed to ARBs [19]. This could be explained by the triggering of a more recently discovered pathway of the RAAS involving the creation of a vasodilatory byproduct, angiotensin-(1-7). Angiotensin-(1-7) is a vasoactive peptide formed from the metabolism of angiotensin I and angiotensin II via angiotensin-converting enzyme-2 (ACE-2), neprilysin, and others. Angiotensin-(1-7) has opposing effects to angiotensin II in that it acts peripherally on Mas receptors to increase vasodilation, diuresis, natriuresis, and anti-fibrotic effects. The upregulation of renin with chronic ARB blockade may feed into the production of angiotensin-(1-7) in some patients and decrease the blood pressure-raising capacity of exogenous AT2S in patients on ARB therapy. Additionally, AT2S effects on the angiotensin II type-2 receptor have counterregulatory effects on the vasoconstriction actions of the angiotensin II type-1 receptor. When ARBs selectively block angiotensin II type-1 receptors, they may offset the impact of exogenous AT2S by increasing angiotensin II type-2 receptor effects [18]. While the above impacts are expected, the utilization of pharmacologic AT2S rapidly restores renin and angiotensin I balance. It is unknown in our patient cohort what the patients’ RAAS profiles were ahead of their DDKT. Perhaps using RAAS profiling (renin, angiotensin I, or angiotensin II serum concentrations) could be advantageous to optimize vasopressor selection in the setting of kidney transplant, particularly in those patients who are on agents that disrupt the RAAS. If renin or angiotensin I levels are high, AT2S could be a preferential agent to restore RAAS balance, and utilization could be guided not only by hemodynamics but also by RAAS parameter normalization. Further studies may warrant exploration.

Lastly, a reason for the potential absence of observed hemodynamic differences between ARB and non-ARB groups could be that the effects of the ARB had worn off by the time of KT. We observed a median cold ischemic time (CIT) of about 14 h in both groups. Assuming that patients were notified of organ availability and told to report to the hospital about 14 h before transplant, the effects of the ARB may have “worn off” if ARB administration had been before the call. Affinity to the angiotensin II type-1 receptor and half-life vary between ARBs, which may also affect AT2S hemodynamic effects [25]. For example, telmisartan has been shown to have a greater affinity to and dissociate slower from the angiotensin II type-1 receptor and contributes to its prolonged half-life (24 h) compared to other ARBs such as losartan and irbesartan, which were used by a majority of our cohort and could have worn off by the time of transplant [18,23,24].

### 4.2. Limitations

There are several limitations in this study. First, the retrospective cohort study design depends on the historical data available in the EMR, which is subject to inaccuracy. This was mitigated by ensuring that consistent data collectors utilized a consistent format. Actual adherence to pre-admission antihypertensive regimens, including the timing of medication administration prior to the start of AT2S, which could impact study outcomes, was not captured. Given the unexpected nature of transplant in DDKT, it is very likely that patients were taking their home medication regimens prior to the notification of organ availability. Furthermore, given that medication nonadherence is a major disqualifier from transplant candidacy, it is again likely that medications were taken as prescribed [26]. Without capturing this exact data point, this will remain a limitation. Second, the small sample size may falsely inflate the size of the observed effects, and we may need more power to capture subtle differences in outcomes. Our sample size was one of convenience, given the utilization of AT2S at our center. Further investigation with a larger sample may be warranted. Third, the chronicity of the pre-admission hypertension was unknown, which could make the observed changes in blood pressure detrimental to outcomes. As hypertension is the leading cause of ESRD in our kidney transplant population, it is likely that these patients were chronically hypertensive. Finally, this study’s external validity is limited due to its single-center nature, especially in institutions that do not yet use AT2S as first-line blood pressure support in the setting of kidney transplant.

## 5. Conclusions

Despite theoretical differences in response, package insert warnings, and observations in other patient populations, no hemodynamic or clinical differences were observed in patients’ response to AT2S for hypotension surrounding kidney transplant in those receiving pre-transplant ARBs. Patients receiving ARBs in this setting may be expected to require slightly higher doses of AT2S, but the duration is likely to be similar. AT2S appears to be a safe and effective first-line vasopressor agent regardless of the home antihypertensive regimen when used for patients with hypotension surrounding kidney transplantation.

## Figures and Tables

**Table 1:** Baseline characteristics.

Baseline Characteristics	ARB N = 22	Non-ARB N = 43	*p*-Value
Age, yr (SD)	56.6 (10.9)	53.1 (12.3)	0.267
Female, n (%)	7 (32)	13 (30)	0.896
BMI, kg/m^2^ (SD)	31.1 (5.5)	33.1 (6.7)	0.222
BMI > 35, n (%)	7 (32)	17 (40)	0.542
**Race/Ethnicity**			
African American race, n (%)	5 (23)	18 (42)	0.127
Hispanic ethnicity, n (%)	7 (32)	15 (35)	0.805
**Comorbid Disease States**			
Coronary artery Disease, n (%)	10 (45)	18 (42)	0.782
Diabetes mellitus, n (%)	13 (59)	23 (53)	0.667
Hyperlipidemia, n (%)	8 (36)	17 (40)	0.840
Hypertension, n (%)	22 (100)	43 (100)	1
**Kidney Transplant-Specific Characteristics**			
DDKT, n (%)	22 (100)	43 (100)	1
Donor age, yr (SD)	44.3 (13.1)	38.6 (13.1)	0.101
Donor terminal Scr, mg/dL (SD)	1.15 (0.61)	1.10 (0.48)	0.69
CIT, h (SD)	14.0 (2.9)	14.5 (3.5)	0.665
KDPI (SD)	57.4 (26.2)	45.6 (23.2)	0.069
Duration of KT surgery, h (SD)	3.2 (5.4)	5.2 (2.1)	0.029
First Post-op SCr, mg/dL (SD)	9.38 (3.39)	8.51 (3.46)	0.338
First Post-op CrCl, mL/min (SD)	6.23 (2.6)	7.63 (3.9)	0.137
**Antihypertensive Characteristics**			
>1 anti-HTN meds, n (%)	17 (77)	25 (58)	0.127
Number of anti-HTN meds, n (IQR)	3.4 (3)	1.8 (2)	0.001
**Antihypertensive Agents**			
ACEi, n (%)	0 (0)	16 (37)	0.017
Calcium channel blocker, n (%)	16 (73)	13 (30)	0.001
Beta blocker, n (%)	15 (68)	30 (70)	0.896
Loop diuretic, n (%)	10 (45)	11 (26)	0.105
Alpha agonist, n (%)	2 (9)	3 (7)	0.762
Direct vasodilators, n (%)	7 (32)	8 (19)	0.232

All data is presented as count n (%) or average (standard deviation, SD) unless otherwise noted. ACEi = angiotensin-converting enzyme inhibitor; ARB = angiotensin II type-1 receptor-blocking agent; BMI = body mass index; CIT = cold ischemic time; CrCl = creatinine clearance; h = hours; HTN = hypertension; KDPI = kidney profile donor index; KT = kidney transplantation; Post-op = postoperative; SCr = serum creatinine; yr = years.

**Table 2:** Hemodynamic outcomes.

Baseline Hemodynamics	ARB N = 22	Non-ARB N = 43	*p*-Value
SBP baseline, mmHg (SD)	151.8 (13.7)	152.8 (24.8)	0.828
DBP baseline, mmHg (SD)	76.0 (10.4)	82.4 (15.4)	0.086
MAP baseline, mmHg (SD)	101.3 (9.9)	105.9 (16.9)	0.245
HR baseline, bpm (SD)	72.5 (9.7)	80.5 (13.5)	0.037
**AT2S Characteristics**			
AT2S max dose, ng/kg/min (SD)	30.0 (21.1)	19.3 (12.3)	0.056
AT2S duration, h (SD)	13.4 (15.1)	26.8 (35.1)	0.267
AT2S duration intraop, h (SD)	1.63 (1.25)	1.93 (1.67)	0.463
AT2S duration postop, h (SD)	14.3 (20.8)	25.6 (36.0)	0.181
**Hemodynamic Outcomes**			
Time w/SBP < 120 mmHg Intraop, h (SD)	1.37 (0.93)	1.27 (1.1)	0.712
Time w/SBP < 120 mmHg Postop, h (SD)	1.15 (1.6)	1.55 (2.0)	0.417
Total time w/SBP < 120 mmHg, h (SD)	2.52 (2.1)	2.82 (2.5)	0.632
# Instances of SBP < 120 mmHg Intraop (SD)	5.5 (3.7)	5.1 (4.4)	0.712
# Instances of SBP < 120 mmHg Post-op (SD)	4.6 (6.3)	6.2 (8.1)	0.417
Second vasopressor needed, n (%)	7 (22)	16 (37)	0.667

All data is presented as count n (%) or average (standard deviation, SD) unless otherwise noted. ARB = angiotensin II type-1 receptor-blocking agent; AT2S = synthetic angiotensin II; DBP = diastolic blood pressure; HR = heart rate; h = hours; MAP = mean arterial pressure; SBP = systolic blood pressure; # = number.

**Table 3:** Intraoperative characteristics.

Push-Dose Vasopressor Usage	ARB N = 22	Non-ARB N = 43	*p*-Value
≥1 push-dose vasopressor(s), n (%)	15 (68.2)	37 (86)	0.880
≥2 push-dose vasopressors, n (%)	6 (27)	16 (37)	0.423
≥3 push-dose vasopressors, n (%)	2 (9)	4 (9)	0.978
**Push-dose Vasopressor Type**			
Phenylephrine, n (%)	11 (50)	25 (58)	0.532
Norepinephrine, n (%)	8 (36)	21 (49)	0.338
Epinephrine, n (%)	4 (18)	7 (16)	0.846
Vasopressin, n (%)	0 (0)	4 (9)	0.023
**Push-dose Vasopressor Dosage**			
Phenylephrine, mcg (SD)	295 (217)	356 (250)	0.483
Norepinephrine, mcg (SD)	34 (15)	47 (44)	0.435
Epinephrine, mcg (SD)	30 (14)	43 (70)	0.732
Vasopressin, units (SD)	0	18 (14)	<0.001
**Sedation**			
Propofol continuous infusion, n (%)	6 (27.3)	16 (37.2)	0.423
Received propofol bolus, n (%)	22 (100)	42 (97.7)	0.471
Total propofol bolus, mg (SD)	138 (36.9)	149 (42.3)	0.324
**Fluid balance**			
Total fluid volume received, mL (SD)	3606 (964)	3382 (1236)	0.461
Estimated blood loss, mL (SD)	177 (322)	153 (255)	0.741
Received blood products, n (%)	16 (72.7)	25 (58.1)	0.249
Blood product volume, mL (SD)	663 (340)	581 (371)	0.479

All data is presented as count n (%) or average (standard deviation, SD) unless otherwise noted. ARB = angiotensin II type-1 receptor-blocking agent.

**Table 4:** Length-of-stay outcomes.

Length-of-Stay Outcomes	ARB N = 22	Non-ARB N = 43	*p*-Value
ICU LOS, days (SD)	4.5 (1.6)	5.1 (1.7)	0.230
Hospital LOS, days (SD)	6.7 (2.7)	6.6 (2.1)	0.866

ARB = angiotensin II type-1 receptor-blocking agent; ICU = intensive care unit; LOS = length of stay.

## Data Availability

Due to restrictions from our ethics office, data from this investigation is unavailable for distribution or sharing.

## Abbreviations

The following abbreviations are used in this manuscript:

ACEis	Angiotensin-converting enzyme inhibitors
AKI	Acute kidney injury
ARBs	Angiotensin receptor-blocking agents
AT2S	Synthetic angiotensin II
BBs	Beta blockers
BMI	Body mass index
BP	Blood pressure
Bpm	Beats per minute
CCBs	Calcium channel blockers
CIT	Cold ischemic time
CrCl	Creatinine clearance
CT	Computed tomography
DA	Dopamine
DBP	Diastolic blood pressure
DDKT	Deceased-donor kidney transplant
DGF	Delayed graft function
DVT	Deep vein thrombosis
EKG	Electrocardiogram
EMR	Electronic medical record
EPI	Epinephrine
ESRD	End-stage renal disease
HR	Heart rate
Hr	Hour
HTN	Hypertension
ICU	Intensive care unit
IQR	Interquartile range
IV	Intravenous
K	Potassium
KDPI	Kidney donor profile index
KT	Kidney transplant
LOS	Length of stay
MAP	Mean arterial pressure
NE	Norepinephrine
PE	Phenylephrine
Post-op	Postoperative
RAAS	Renin–angiotensin–aldosterone system
SBP	Systolic blood pressure
Scr	Serum creatinine
SD	Standard deviation
SGF	Slow graft function
